# Relationship between media multitasking and functional connectivity in the dorsal attention network

**DOI:** 10.1038/s41598-020-75091-9

**Published:** 2020-10-22

**Authors:** Kei Kobayashi, Naoya Oishi, Sayaka Yoshimura, Tsukasa Ueno, Takashi Miyagi, Toshiya Murai, Hironobu Fujiwara

**Affiliations:** 1 Department of Neuropsychiatry, Faculty of Medicine, Kyoto University, Kyoto, Japan; 2 Medical Innovation Center, Kyoto University Graduate School of Medicine, Kyoto, Japan; 3 Department of Neurodevelopmental Psychiatry, Habilitation and Rehabilitation, Kyoto University, Kyoto, Japan; 4 Integrated Clinical Education Center, Kyoto University Hospital, Kyoto, Japan; 5 Artificial Intelligence Ethics and Society Team, RIKEN Center for Advanced Intelligence Project, Tokyo, Japan

**Keywords:** Cognitive neuroscience, Human behaviour

## Abstract

With the development of digital technology, media multitasking behaviour, which is using two or more media simultaneously, has become more commonplace. There are two opposing hypotheses of media multitasking with regard to its impact on attention. One hypothesis claims that media multitasking can strengthen attention control, and the other claims heavy media multitaskers are less able to focus on relevant tasks in the presence of distractors. A total of 103 healthy subjects took part in this study. We measured the Media Multitasking Index (MMI) and subjects performed the continuous performance test. Resting state and oddball task functional MRI were conducted to analyse functional connectivity in the dorsal attention network, and the degree centrality (DC) was calculated using graph theory analysis. We found that the DCs in the dorsal attention network were higher during resting state than during the oddball task. Furthermore, the DCs during the task were positively correlated with the MMI. These results indicated that the DC reduction from resting state to the oddball task in high media multitaskers was attenuated compared with low media multitaskers. This study not only reveals more about the neurophysiology of media multitasking, but could also indicate brain biomarkers of media multitasking behaviour.

## Introduction

The development of digital technology has not only changed our lives, but has also affected our cognitive functioning. In particular, adolescents and young adults tend to use various types of media simultaneously, which is defined as media multitasking behaviour^1^. The accumulation of media multitasking has raised concerns about its impact on attention, and has given rise to two opposing hypotheses^2^. The scattered attention hypothesis claims that long-term media multitasking weakens attention control; that is, individuals who have been exposed to a multitasking lifestyle are less able to maintain focus on relevant tasks in the presence of distractors^3^. In contrast, the trained attention hypothesis claims that frequent multitasking practice strengthens cognitive control and positively affects attention^4^. Although several studies have reported the association between media multitasking and attention, the results have been inconsistent.

The Media Multitasking Index (MMI)^3^ or its adapted version^5^ are representative indexes to estimate the effect of media multitasking. A recent longitudinal study reported that younger adolescents with higher MMI (i.e., a stronger tendency towards media multitasking) exhibited more attention problems^6^.

Behavioural investigations into the relationship between media multitasking and attention are generally classified into two categories depending on which aspect of attention is studied—switching capacity or focused attention. Switching capacity, or divided attention, is the ability to shift attention between a small set of cognitive tasks. Considering that media multitasking behaviour generally involves continual switching of attention between multiple media sources, these studies have investigated the impact of a multitasking lifestyle on multitasking ability itself. In accordance with the two opposing hypotheses on attention, the results have not been unanimous. For example, some studies have reported that there is a greater processing cost of switching between task sets in heavy media multitaskers^3,7^, which supports the scattered attention hypothesis. In contrast, one study reported that the degree of media multitasking was associated with a better task switching performance^4^, which supports the trained attention hypothesis.

Similarly, inconsistent findings have been reported for focused attention, which is the ability to ignore distractors. In the studies that investigated correlations between focused attention and multitasking, several studies suggested that heavy multitaskers were less capable of focused attention processing than light multitaskers^3,8,9^. However, other studies have failed to find significant differences in focused attention between heavy and light media multitaskers^10,11^. These discrepancies might be caused by differences in the types of media or the nature of media multitasking, as a result of rapidly changing modern lifestyles. However, two major factors should also be considered to explain the discrepancies, as follows. The first involves the possible insufficiency of statistical power in some attention paradigms. A meta-analysis suggested that the association between media multitasking and distractibility is weak, and is strongly influenced by small-study effects^12^. The other possible cause of discrepancies in the literature involves differences in the degree of multitasking in the investigated subjects across studies. Interestingly, one study reported that intermediate media multitaskers performed better on attentional tasks than both light and heavy media multitaskers^13^. This finding suggests a possible U-shaped association between focused attention and media multitasking, which might account for the inconsistencies among the results of previous studies.

In the present study, we attempted to further explore the relationship between focused attention and multitasking lifestyles. Considering the aforementioned potential sources of discrepancies in the literature, we planned our investigation as follows. First, we conducted functional MRI (fMRI) scans to assess brain activity associated with focused attention, expecting to detect neural correlates of multitasking tendencies even if we failed to discover any associations in the behavioural data. Second, considering the possibility of a non-linear association between media multitasking lifestyles and focused attention, we focused on a narrow range of target subjects in terms of their multitasking level; that is, we concentrated on normal healthy subjects who were presumed to have low to intermediate multitasking levels.

A few neuroimaging studies have examined the neural correlates of media multitasking^5,14^, and these have found that the anterior cingulate cortex, precuneus, and prefrontal regions are associated with media multitasking. No studies have investigated the large-scale brain networks that underlie media multitasking, yet the concept of large-scale networks is important to understand brain network properties and the organisation of cognitive functions^15^. Among multiple large-scale networks, we paid special attention to the dorsal attention network (DAN) because the DAN is a representative large-scale network that is activated during attention tasks, such as auditory oddball tasks^16^. The DAN is also responsible for focused attention and goal-directed top-down attentional processing^17,18^. Insufficient recruitment of the DAN can lead to failures in maintaining goal-directed attention, which impairs task performance^19^. As a marker of intra-network connectivity in the DAN, we used degree centrality (DC), which is defined as the number of functional connectivities (FCs) in which connectivity strength surpasses a certain threshold. The DC is a representative parameter of graph theory analyses. Furthermore, as supplementary analyses, we also investigated the ventral attention network (VAN), salience network (SN), and frontoparietal network (FPN), which are all known to be related to attention^20,21^.

In the main analyses, we compared the DCs of the DAN during resting state and during a task that demanded focused attention. We then computed the correlations between MMI and the DC during both resting state and the task. It has been reported that smaller differences in FC patterns from resting state to task state within large-scale networks are associated with better cognitive function^22^. Based on this previous study, our tentative assumption for the controversy around the scattered attention vs. trained attention hypotheses was as follows. If our data showed smaller differences in DCs between the task and resting conditions in higher multitaskers than in lower multitaskers, they would support the trained hypothesis; that is, multitasking habits in the former group may have fostered a homeostatic activation pattern in the DAN in response to attentional demand. However, if our data showed the opposite results, they would support the scattered attention hypothesis.

## Results

### The media multitasking index and task performance

**Figure 1:**
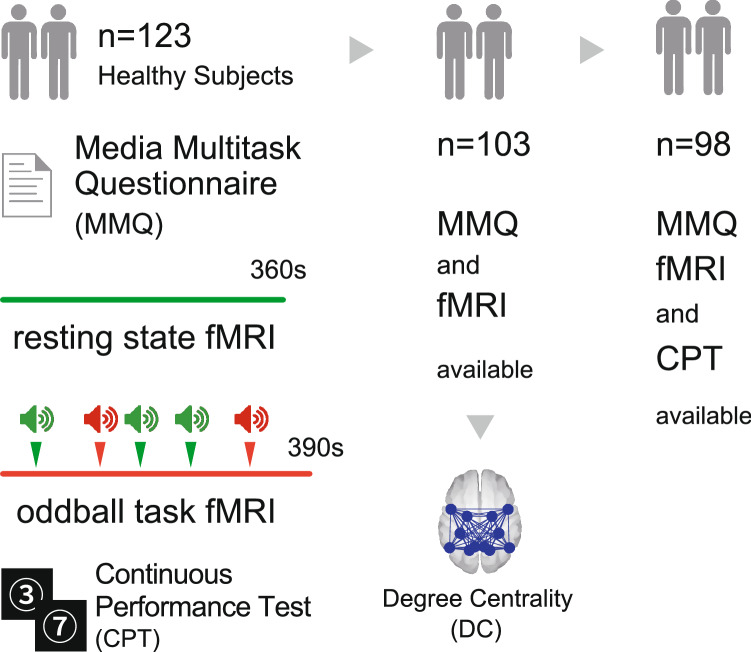
Research design. A total of 123 subjects performed all tests; 20 subjects were excluded due to head motion and incomplete questionnaires. Degree centrality of the dorsal attention network was calculated from this final total of 103 subjects. Another five subjects were excluded due to errors during the continuous performance test.

We recruited 123 subjects. However, 19 subjects were excluded because of head movements during fMRI, and 1 subject was excluded due to an incomplete questionnaire. Finally, 103 subjects (66 male, 29 ± 11.6 years) were included in the present analysis (Fig. 1).

To estimate focused attention performance, participants completed a visual continuous performance test (CPT) outside the scanner, in addition to the auditory oddball task during fMRI acquisition. Table 1 displays demographic data, the MMI, CPT scores, and oddball task scores. The mean MMI was 1.68 (SD = 1.07), which was lower than the mean scores from previous studies (cf. Ophir et al., 2009: mean MMI = 4.38 ± 1.52; Alzahabi and Becker, 2013: mean MMI = 4.07 ± 1.64; Cardoso-Leite et al., 2016: mean MMI = 3.98 ± 1.99)^3,4,13^. The MMI in the present study was distributed from light- to intermediate-level multitasking, as defined by Cardoso-Leite et al.^13^.

**Table 1:** Demographic and behavioural data.

Variables	Mean	SD
n	103	
Age (years)	29.0	11.6
Sex (male/female)	66/37	
MMI	1.7	1.1
RT_CPT (ms)_	417.2	69.0
CV_CPT_	15.8	6.0
RT_odd (ms)_	405.3	77.6
CV_odd_	17.8	9.9

*MMI* Media Multitasking Index, *CPT* continuous performance test, *odd* oddball task fMRI, *RT* reaction time, *CV* coefficient of variation.

Among the 103 subjects, those who had fewer than 75% valid responses on the CPT were excluded from the CPT analysis (N = 5; Fig. 1). We calculated the reaction time (RT_CPT_) and coefficient of variation (CV_CPT_) from the CPT performances. No significant association was found between the MMI and age, or between the MMI and any CPT parameters. We also calculated the RT_odd_ and CV_odd_ from the oddball task results, and these were not correlated with the MMI (Table 2).

**Table 2:** Correlations between MMI and age, CPT, and oddball parameters.

		Age (years)	RT_CPT_	CV_CPT_	RT_odd_	CV_odd_
MMI	Correlation coefficient	− 0.11	− 0.07	0.12	− 0.54	− 0.01
	p (two-sided)	0.28	0.47	0.23	0.60	0.95

The correlation coefficient was calculated using Spearman’s rank correlation.

*MMI* Media Multitasking Index, *CPT* continuous performance test, *odd* oddball task fMRI, *RT* reaction time, *CV* coefficient of variation.

### Differences in degree centralities between resting-state and oddball task fMRI

**Figure 2:**
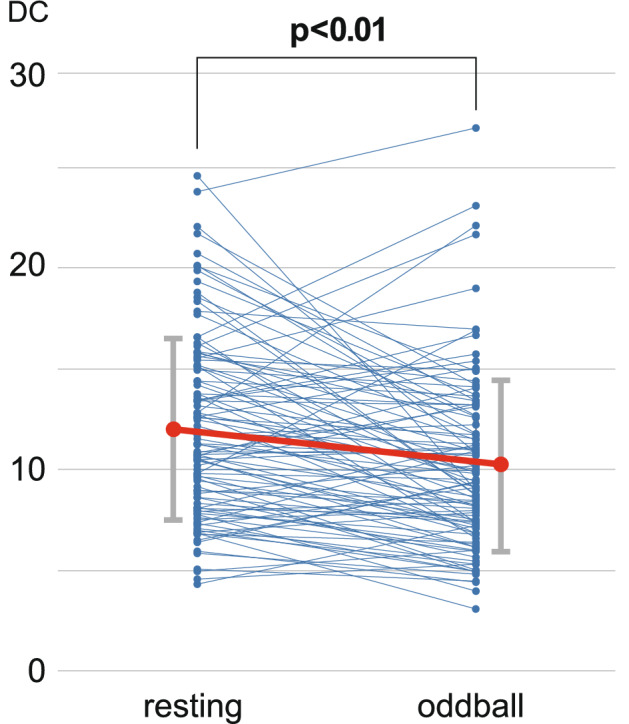
Difference in DCs between resting-state and oddball task fMRI. Each dot indicates the value of degree centrality (DC) of 103 subjects. There was a significant decrease of DCs from resting state to oddball task fMRI, as calculated using paired t-tests (t = 4.56, p < 0.01).

To estimate the topological properties of the DAN, we analysed FCs using fMRI data obtained from each subject. We also calculated DC of FCs using graph theory analysis, which reflects the density of FCs in the DAN. The area under the curve, which is the sum of the DCs of the defined thresholds, was adopted as the main parameter of the analysis. The anatomical names and peak coordinates of the DAN are shown in Table 2. A paired t-test showed that the DCs across the DAN during resting state were significantly higher than those seen during the oddball task (df = 103, t = 4.53, Cohen’s d = 0.45, p < 0.001; Fig. 2).

**Figure 3:**
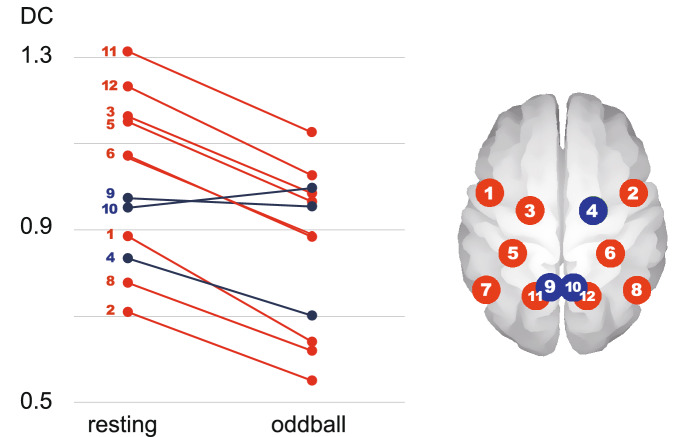
Differences in DCs in each ROI between resting-state and oddball task fMRI. Each dot indicates the averaged degree centrality (DC) of each region of interest (ROI) in the dorsal attention network. The dots and ROIs in red show significant DC differences between the resting state and oddball task. Those in blue did not show any significant differences.

Furthermore, we compared the DCs of each region of interest (ROI) during resting state and the oddball task. The DCs at resting state were significantly larger than those seen during the oddball task in 9 out of 12 ROIs, including the left superior frontal gyrus (df = 103, t = 3.34, d = 0.33, p = 0.001), bilateral superior parietal lobules (including four ROIs: R, df = 103, t = 3.71, d = 0.36, p < 0.001 and df = 103, t = 3.66, d = 0.36, p < 0.001 ; L: df = 103, t = 3.92, d = 0.39, p < 0.001 and df = 103, t = 4.35, d = 0.43, p < 0.001), bilateral inferior temporal gyri (R: df = 103, t = 3.53, d = 0.35 , p < 0.001 ; L: df = 103, t = 3.21, d = 0.31, p = 0.002), and bilateral precentral gyri (R: df = 103, t = 5.40, d = 0.53 p < 0.001 ; L: df = 103, t = 3.83, d = 0.38, p < 0.001). Another three ROIs did not show significant differences between the two conditions, including the bilateral precunei (R: df = 103, t = − 0.90, d = 0.04, p = 0.37; L: df = 103, t = 0.41, d = − 0.09, p = 0.69) and right superior frontal gyrus (df = 103, t = 2.17, d = 0.21, p = 0.03) (Fig. 3).

### Correlations between the media multitasking index and degree centralities

**Figure 4:**
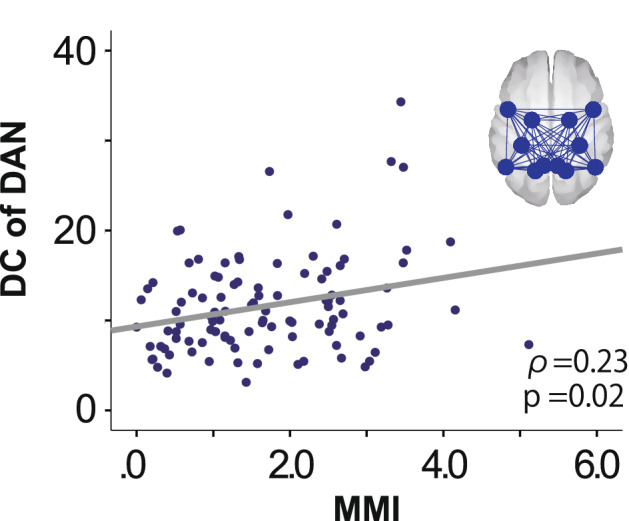
Correlation between MMI and DCs of the DAN during the oddball task. The correlation coefficient ρ was calculated using Spearman’s rank correlation. *MMI* Media Multitasking Index scores, *DC* degree centrality, *DAN* dorsal attention network.

There was no significant correlation between the MMI and DCs during resting state (df = 103, ρ = 0.14, p = 0.16). However, there was a significant association between the MMI and DCs across the DAN during the oddball task (df = 103, ρ = 0.23, p = 0.02; Fig. 4). We also calculated the partial correlations between the MMI and DCs with age and CPT parameters (df = 91, ρ = 0.20, p = 0.049) and age and oddball parameters (df = 98, ρ = 0.21, p = 0.048) as covariates. The correlation between the MMI and DCs remained significant after controlling for these variables.

**Figure 5:**
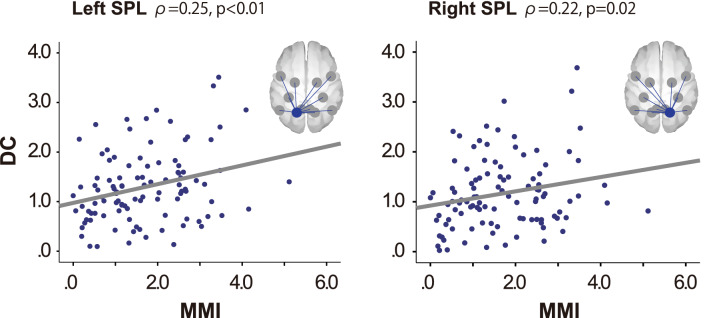
Correlation between the MMI and DCs of the bilateral superior parietal lobule during the oddball task. The correlation coefficient was calculated using Spearman’s rank correlation. *SPL* superior parietal lobule, *MMI* Media Multitasking Index scores, *DC* degree centrality.

Furthermore, we calculated the correlations between the MMI and DCs of each ROI during the oddball task. The MMIs were significantly correlated with the DC of the left superior parietal lobule (df = 103, ρ = 0.27, p = 0.006) and the right superior parietal lobule (df = 103, ρ = 0.22, p = 0.02), which are located in the bilateral superior parietal lobule (Fig. 5).

### Functional connectivity analyses in other attention-related networks

As supplementary analyses, we further examined the associations between media multitasking and FCs in three other attention-related networks: the VAN, the SN, and the FPN. Details are given in the Supplementary Information.

## Discussion

This is the first study to investigate the neural correlates of intermediate media multitasking tendencies in the context of the focused attention network. In this area of research, there is ongoing debate as to whether the scattered attention hypothesis or the trained attention hypothesis best reflects the effect of media multitasking on attention. By investigating the FCs within the DAN both during rest and focused attention, our results shed light on the controversy between these two opposing hypotheses, and the changes in FCs from resting state to the task phase and their association with the MMI tentatively support the trained attention hypothesis.

The mean MMI was lower than the mean scores from previous studies^3,4,13^ and was distributed from a light to intermediate level of multitasking^13^. We found no significant correlation between the MMI and CPT performance. Across subjects, the averaged DCs throughout the DAN during the oddball task were smaller than those during resting state. Most importantly, the MMI was positively correlated with DCs of the DAN during the oddball task.

There was no significant correlation between media multitasking and the behavioural performance of focused attention. Our results might be due to the smaller number of subjects relative to these previous studies.

We found reduced DCs during the oddball task compared with DCs during rest. Few studies have investigated differences in FCs between resting state and during attention tasks. However, Tomasi et al. found that, during a simple cognitive task, the FC density, which is similar to DC in our study, was significantly lower than that during resting state in the visual, auditory, language, somatosensory, and motor/premotor cortices^23^. The authors claimed that the ability to reduce connectivities might be crucial to engage in cognitive tasks.

Our main finding was that there was a positive correlation between the MMI and DCs during the oddball task. In other words, the DC reduction from resting state to the oddball task was attenuated in higher media multitaskers compared with lower media multitaskers. A recent fMRI study suggested that higher performance in a cognitive task is associated with less reconfiguration of the related large-scale network during the task (i.e., smaller differences in FC patterns between resting and task conditions)^22^. If we apply this idea to the interpretation of our current results, smaller DC differences in the higher multitaskers suggest that their DANs were more fit for the demand of focused attention. We could further speculate that the DANs of the higher multitaskers in our subjects had been more “trained” during their past multitasking.

As mentioned in the introduction, one study has reported that intermediate multitaskers have the best performance on attention tasks, which indicates that the relationship between media multitasking and attention can be modelled with an inverted U-shape curve^13^. If we accept this model, most of the subjects in our study are located on the left shoulder of the inverted U, as their MMIs ranged from low to intermediate levels. Therefore, the positive correlation found between the MMI and DCs during the oddball task could indicate that higher DCs in intermediate multitaskers result in better attention performance. This interpretation supports the trained attention hypothesis, but only for individuals within the range of light to intermediate multitaskers.

As exploratory analyses, we also investigated the difference in DCs between each ROI at resting state and during the oddball task. Although DCs in almost all ROIs during resting state were larger than those during the oddball task, an exception was found in the bilateral precuneus. The precuneus, as a part of the DAN, is known to play a role in focused attention as well as shifting attention^24^. Furthermore, the precuneus is known to be the core region of the default mode network^25^. Its role as a “hub” between the DAN and the default mode network could explain why a different activation pattern was noted in the precuneus during our experimental paradigm, compared with other regions of the DAN.

We also investigated the relationship between DCs and MMIs within each ROI. DCs of the bilateral superior parietal lobule during the oddball task were positively correlated with the MMI. This means that the DC reduction in the superior parietal lobule was smaller in high media multitaskers than low ones. Posner et al. found that superior parietal lobule lesions led to a deficit in disengaging attention^26^. Moreover, fMRI studies have suggested that the superior parietal lobule is the source of a brief attentional control signal to shift the attentive state^27^. Therefore, the task-associated DAN changes in high media multitaskers could be partially associated with the disengaging and shifting attention function.

As supplementary analyses, we further examined the associations between media multitasking and the other attention-related networks—namely, the VAN, SN, and FPN—because media multitasking involves multiple attention properties and may be influenced by the interactions of various brain networks^20,21^. First, there were significant reductions in the DCs from rest to task performance in each of the networks (Table S1). These results were in line with a previous study that suggested that FC density during cognitive tasks with relatively low cognitive loads is decreased compared with FC density during resting state^23^. Second, there were significant correlations between the DCs of the DAN and those of the VAN during resting state as well as the oddball task (Table S2). These results suggest that these two attentional networks have a higher tendency of covariation, both at rest and during the task. Finally, regarding the relationship between DCs and MMI, there were no significant correlations in the VAN, SN, or FPN (Table S3), which was different from the results of the DAN. Thus, among the attention-related networks, the DAN seems to be the most affected by media multitasking tendencies.

The current study has several limitations that should be considered. First, and most importantly, the correlations between MMI and the attentional tasks were not significant in the current study. Therefore, for the observed correlations between DC and MMI, the inferences about the underlying mechanisms that are discussed in the fifth paragraph of the discussion should be considered tentative, rather than conclusive. In future studies, one possibility to overcome this shortcoming would be to apply attentional tasks that have a higher cognitive load and greater variance among subjects. Second, the MMI includes the use of various media, such as television, computer-based video, social network services, video games, and E-mail. In the current study, it is unclear which media most impacted the results. The use of each medium should be investigated separately and the results should be compared between various media in future studies. Third, we could not conclude whether the media multitasking behaviour was beneficial due to the cross-sectional study design. Thus, we could not settle the controversy between the scattered attention hypothesis and the trained attention hypothesis. A longitudinal follow-up study is necessary to clarify the causal relationship between media multitasking and focused attention. Fourth, in our FC analyses, especially during the task, we were unable to discern whether the correlated activity between two brain areas was a “real” neural interaction between two regions, or if it was simply the independent and simultaneous co-activation of the two regions^28^. Finally, our current results should be interpreted with caution because we did not study a full range of psychological and physiological confounding factors that may affect the correlation analyses.

In conclusion, we examined the association between media multitasking and the topological properties of the DAN during a focused attention task for low- to intermediate-level multitaskers, and found that the task-related brain activity in high media multitaskers was similar to that during resting state. These results shed light on the neurophysiological basis of multitasking behaviour. Topological properties of the DAN could be a brain biomarker of media multitasking behaviour, which is an imminent issue in modern society.

## Methods

### Subjects

We recruited 123 right-handed healthy subjects through advertisements and individual contact. No subjects had any psychiatric disorder or severe physical illness. The estimated intelligence quotients (IQs) were measured using the Japanese Version of the Adult Reading Test^29^, and all subjects fell within the normal range.

The study was approved by the Ethics Committee of the Kyoto University Graduate School and Faculty of Medicine and was conducted in accordance with the guidelines of the Declaration of Helsinki. All subjects provided written informed consent.

### The media multitasking questionnaire

We used a modified version of the Media Multitasking Questionnaire (MMQ) translated into Japanese to measure media multitasking activity^5^. The MMQ has two main sections. The first section measures how many hours respondents spend using 12 common media per week. The media types in the modified MMQ were as follows: print media, television, computer-based video (such as YouTube), music, social network services (such as Facebook and Twitter), video or computer games including mobile phone games, telephone and mobile phone voice calls, instant messaging (such as LINE, Facebook messengers), short message service (text messaging), E-mail, web surfing, and other computer-based applications (such as word processing). In the second section, subjects were asked to complete a media multitasking matrix that measures how frequently any of the other types of medium was concurrently used together with the primary medium. Finally, we calculated the Media Multitasking Index (MMI), which is an indication of the level of media multitasking during consumption of any media. The detailed calculation methods and the procedures for making the Japanese version are shown in the Supplementary Information.

### Continuous performance test

We conducted the Continuous Performance Test (CPT) to estimate focused attention at the behavioural level. The CPT was completed on a laptop PC. We adopted the A–X version CPT^30^. In the A–X CPT, a series of random one-digit numbers are displayed 400 times, and the subject is instructed to press the spacebar as quickly as possible when the number “7” is immediately followed by the number “3”. The task length was 16 min 40 s and the target stimuli occurred 10% of the time. The CPT performance was estimated by reaction time (RT_CPT_) and the coefficient of variation (CV_CPT_).

### MRI acquisition

Functional MRI (fMRI) acquisition was performed during two consecutive conditions. The first condition was a 360-s resting state scan, and the second was a 390-s auditory oddball task scan. We used a single-shot gradient-echo echo planar imaging pulse sequence on a 3-T MRI unit (Tim-Trio; Siemens, Erlangen, Germany) with a 40-mT/m gradient and a receiver-only 32-channel phased-array head coil. During the resting state condition, participants were instructed to look at the cross that appeared on the monitor without thinking about anything specific. Subsequently, during the oddball task condition, the subjects heard either the target or non-target stimuli, and were required to press a button as quickly and accurately as possible when they heard the target stimuli. Thirty target sounds and 150 non-target sounds were presented in 390 s. Additional details of the MRI acquisition are provided in the Supplementary Information.

### Image preprocessing

Resting state and oddball task fMRI datasets were corrected for EPI distortion using FMRIB’s Utility for Geometrically Unwarping EPIs (FUGUE), which is a part of the FSL software package (FMRIB’s software library, ver. 5.0.9; https://www.fmrib.ox.ac.uk/fsl). Artifact components and motion-related fluctuations were then removed from the images using FMRIB’s ICA-based X-noiseifier (FIX)^31^. After preprocessing, the structural and functional MRI data were statistically analysed using the CONN-fMRI Functional Connectivity toolbox (ver. 17e; www.nitrc.org/projects/conn) combined with the statistical parametric mapping software package SPM12 (Wellcome Trust Centre for Neuroimaging; https://www.fil.ion.ucl.ac.uk/spm).

All functional images were initially realigned and unwarped, slice-timing corrected, co-registered with structural data, spatially normalised into the standard MNI space (Montreal Neurological Institute, Montreal, Canada), outlier detected (ART-based scrubbing), and smoothed using a Gaussian kernel with a full-width-at-half maximum (FWHM) of 8 mm. All preprocessing steps were conducted using a default preprocessing pipeline for volume-based analysis (to MNI space). Structural data were segmented into grey matter, white matter (WM), and cerebrospinal fluid (CSF), and normalised in the same default preprocessing pipeline. Principal components of signals from WM and CSF, as well as translational and rotational movement parameters (with another six parameters representing their first-order temporal derivatives), were removed using covariate regression analysis by CONN. Using the implemented CompCor strategy^32^, the effect of nuisance covariates, including fluctuations in fMRI signals from WM, CSF, and their derivatives, as well as realignment parameter noise, were reduced. As recommended, band-pass filtering was performed with a frequency window of 0.008–0.09 Hz. This preprocessing step was found to increase retest reliability. We did not remove mean evoked responses prior to task-state FC analysis (Cole et al. 2019).

To evaluate head movement during fMRI, we used framewise displacement, which quantifies head motion between each volume of functional data^33^. Subjects were excluded if the number of volumes in which head position was 0.5 mm different from adjacent volumes was more than 20%^34^.

### Functional connectivity and graph theory analysis

We conducted a region of interest (ROI)-to-ROI functional connectivity (FC) analysis using the CONN toolbox. We specified 12 spherical clusters of the dorsal attention network (DAN) with 10-mm diameters and peak coordinates based on a previous fMRI study^35^ (Table 3). For each subject, preprocessed blood oxygenation level-dependent (BOLD) time series of all voxels in each ROI were averaged. The FC was computed using the Fisher-transformed bivariate correlation coefficients between two ROIs BOLD time series. All pairs of ROIs constructed a 12 × 12 FC matrix for each subject. We also conducted ROIs of the ventral attention network (VAN), salience network (SN), and frontoparietal network (FPN), which are associated with other aspects of attention. More details are provided in the Supplementary Methods.

**Table 3:** Coordinates of regions of interest in the dorsal attention network.

Region of interest	X	Y	Z
Superior frontal gyrus	± 22	− 8	54
Superior parietal lobule	± 34	− 38	44
Superior parietal lobule	± 18	− 69	51
Inferior temporal gyrus	± 51	− 64	-2
Precuneus	± 8	− 63	57
Precentral gyrus	± 49	3	34

These 12 regions of interest of the dorsal attention network were obtained from Yeo et al., 2010. The peak coordinates were based on the atlas space of the Montreal Neurological Institute.

Among the strategies for the FC analyses, graph theory is one that can be applied to estimate topological properties of global and local networks using the combination of nodes and edges^36^. We adopted the degree centrality (DC) for our analyses, as the DC is the most basic measure of graph theory analyses, and several studies have suggested that the DC in large-scale networks is associated with the performance of attention-related tasks^23,37^. To calculate DC, FCs within any pairs of ROIs were converted to bivariate undirected edges that were thresholded by the value of the correlation coefficients (CC) of FCs. The threshold range was 0.15 ≤ CC ≤ 0.60 (0.01 step). The upper limit of this threshold value was determined according to a previous study^23^. Then, the number of edges was calculated for each ROI of each individual, for each threshold value. The number of edges was plotted against thresholds, and the area under the curve was calculated and considered as a summarized scalar reflecting the DC of each ROI. The sum of DCs of each ROI (divided by two) was considered as the DC of the entire network.

### Statistical analyses

First, we investigated the correlation of the MMI with age, CPT parameters, and oddball task performance (RT and CV). A one-sample Kolmogorov–Smirnov test revealed that the MMIs, CPT parameters, and oddball task results were not normally distributed. Consequently, Spearman’s rank correlation was used for all the correlation analyses. The correlation was considered significant at p < 0.05.

Second, to compare the DCs between the resting state and oddball task state, paired t-tests were applied to the mean value of DCs in the entire DAN, as well as to the DCs in each ROI. A p-value of < 0.05 was considered to indicate a significant difference. We also calculated the effect size in Cohen’s d. Multiple comparisons correction was applied for the ROI-specific DCs (the Bonferroni corrected p-value was 0.05/12).

We then calculated the correlation of MMI scores with the DCs at resting state and during the oddball task. We also calculated the partial correlation coefficient whilst controlling for age and CPT parameters as confounding variables. A p-value < 0.05 was considered significant. Owing to the exploratory nature of this part of the study, correction for multiple comparisons was not applied for the correlation analysis between MMIs and the DCs in each ROI.

As supplementary analyses, we further examined the associations between media multitasking and the other attention-related networks; namely, the VAN, SN, and the FPN. The results are shown in the Supplementary Information.

## Supplementary information

Supplementary Information.

## Data Availability

The datasets generated and analysed during the current study are available from the corresponding author upon reasonable request.
