# Supplementary material for: Relationship between media multitasking and functional connectivity in the dorsal attention network
Source: Sci Rep. 2020 Oct 22;10:17992. doi: 10.1038/s41598-020-75091-9 (PMC7582949; doi:10.1038/s41598-020-75091-9)
Supplement: Supplementary file 1 — Supplementary Information. [file 41598_2020_75091_MOESM1_ESM.docx]

Supplementary Information

**Relationship between media multitasking and functional connectivity in the dorsal attention network**

Kei Kobayashi^1^, Naoya Oishi^2^, Sayaka Yoshimura^3^, Tsukasa Ueno^1,4^, Takashi Miyagi^1^, Toshiya Murai^1^, Hironobu Fujiwara^1,5^*

^1^Department of Neuropsychiatry, Faculty of Medicine, Kyoto University, Kyoto, Japan

^2^Medical Innovation Center, Kyoto University Graduate School of Medicine, Kyoto, Japan

^3^Department of Neurodevelopmental Psychiatry, Habilitation and Rehabilitation, Kyoto University, Kyoto, Japan

^4^Integrated Clinical Education Center, Kyoto University Hospital, Kyoto, Japan

^5^Artificial Intelligence Ethics and Society Team, RIKEN Center for Advanced Intelligence Project, Tokyo, Japan

*hirofuji@kuhp.kyoto-u.ac.jp

**Supplementary Results**

**Differences in degree centralities between resting-state and oddball task fMRI, for the ventral attention network, salience network, and frontoparietal network**

We further examined the associations between media multitasking and the ventral attention network (VAN), salience network (SN), and frontoparietal network (FPN). As with our main results, we compared the degree centralities (DCs) during resting state with those during the oddball task within each of the VAN, SN, and FPN. Paired t-tests revealed that the DCs during resting state were significantly higher than during the oddball task for all three networks (Table S1).

**Correlations between the degree centralities of the dorsal attention network and other networks**

We investigated the Pearson’s correlations of DCs between the DAN and each of the other networks during resting state as well as during the oddball task. There were significant correlations between the DC of the DAN and that of the VAN during resting state (r = 0.20, p = 0.047) as well as during the oddball task (r = 0.28, p = 0.004) (Tables S2, S3).

**Correlations between the Media Multitasking Index and degree centralities**

We calculated the Spearman’s rank correlations between the Media Multitasking Index (MMI) and the DCs during resting state and the oddball task in the VAN, SN, and FPN. There were no significant correlations between the MMI and DCs during resting state or during the oddball task for the VAN, SN, or FPN (Table S4).

**Supplementary Methods**

**The Media Multitasking Questionnaire**

We used a modified version of the Media Multitasking Questionnaire (MMQ) translated into Japanese to measure media multitasking activity [1]. The MMQ has two main sections. The first section measures how many hours respondents spend using 12 common media per week. In the second section, subjects were asked to complete a media-multitasking matrix that measures how frequently any of the other types of medium was used concurrently with the primary medium. The frequencies were scored as “most of the time” (= 1), “some of the time” (= 0.67), “a little of the time” (= 0.33), or “never” (= 0). The MMI was calculated from the scores of this matrix using the following formula:

$$MMI= \sum_{i=1}^{11} \frac{m_{i}\times h_{i}}{h_{total}}$$

In the formula, m_i_ is the number of media typically used while using the primary medium i, h_i_ is the number of hours per week reportedly spent using the primary medium i, and h_total_ is the total number of hours per week spent using any media. Thus, the index is an indication of the level of media multitasking during consumption of any media.

We created a Japanese version of the MMQ. For this, we obtained permission from the first author of the original version [2] as well as from the first author of the modified version [1]. Next, a qualified clinical psychiatrist and a cognitive science researcher translated the scale into Japanese. The draft of the Japanese version was back-translated by a professional translator. The back-translated MMQ was reviewed by the authors, further revised, and finalised according to their comments.

**MRI Acquisition**

Functional MRI (fMRI) acquisition consisted of two consecutive conditions. The first condition was a 360-second resting-state scan, and the second was a 390-second auditory oddball task scan. We used a single-shot gradient-echo echo planar imaging (EPI) pulse sequence on a 3-Tesla MRI unit (Tim-Trio; Siemens, Erlangen, Germany) with a 40-mT/m gradient and a receiver-only 32-channel phased-array head coil. To minimise head motion during imaging, subjects’ heads were fixed with foam rubber pads. Participants held a button box in their right hand. During the resting state condition, they were instructed to look at the cross that was displayed in the centre of the monitor without thinking about anything specific. Subsequently, the oddball task instructions were presented to subjects both audibly and visually. Subjects heard two types of auditory stimuli in randomised order and pressed a button as quickly and accurately as possible when they heard target stimuli. Subjects were presented with 30 target sounds and 150 non-target sounds in 390 seconds. Non-target stimuli were 150 pure 400-Hz tones and target stimuli were 30 pink noise sounds, which were generated using the software Audacity 2.1.1.3.0 (https://www.audacityteam.org/). All stimuli were presented using E-prime 2.0 software (Psychology Software Tools Inc., Pittsburgh, USA) for 200 milliseconds with a randomised interstimulus interval of 1–3 seconds in 100-millisecond units. Task performance was measured in each subject using the reaction time (RT_odd_) and coefficient of variation (CV_odd_) obtained by pressing the button.

Structural MRI data were also acquired using three-dimensional magnetization-prepared rapid gradient-echo (3D-MPRAGE) sequences. The parameters for the 3D-MPRAGE images were as follows: echo time (TE), 3.4 ms; repetition time (TR), 2000 ms; inversion time, 990 ms; field of view (FOV), 225×240 mm; matrix size, 240×256; resolution, 0.9375×0.9375×1.0 mm^3^; and 208 total axial sections without intersection gaps. Parameters for the fMRI were as follows: TE, 30 ms; TR, 2500 ms; flip angle, 80°; FOV, 212×212 mm; matrix size, 64×64; in-plane spatial resolution, 3.3125×3.3125 mm^2^; 40 total axial slices; and slice thickness, 3.2 mm with 0.8-mm gaps in ascending order. A dual-echo gradient-echo dataset for B0-field mapping was also acquired for distortion correction.

**Functional connectivity and graph theory analysis within the ventral attention network, salience network, and frontoparietal network**

We further examined the associations between media multitasking and the other attention-related networks (namely, the VAN, SN, and FPN) because media multitasking involves multiple attention properties [3,4]. As with the main analyses, we conducted a region of interest (ROI)-to-ROI functional connectivity (FC) analysis using the CONN toolbox. We specified the spherical clusters of the VAN and the FPN with 10-mm diameters and peak coordinates based on a previous fMRI study [5]. The SN was extracted from the functional ROI atlases of the FIND Lab, Stanford University [6]. The ROIs in each network were located as follows: the VAN (12 ROIs), bilateral middle frontal gyrus, bilateral inferior parietal gyrus, bilateral middle temporal gyrus, bilateral cingulate gyrus, bilateral cingulate sulcus, and bilateral insula; the anterior SN (8 ROIs), bilateral middle frontal gyrus, bilateral insula, bilateral medial prefrontal cortex, and bilateral cerebral crus; the posterior SN (12 ROIs), left middle frontal gyrus, bilateral supra marginal gyrus, bilateral precuneus, right middle cingulate cortex, bilateral thalamus, bilateral cerebellum, and bilateral posterior insula; and the FPN (12 ROIs), bilateral middle frontal gyrus, bilateral inferior parietal lobule, bilateral middle temporal gyrus, bilateral medial aspect of the frontal lobe, bilateral cingulate gyrus, and bilateral superior parietal lobule. For each subject, the preprocessed blood oxygenation level-dependent (BOLD) time series of all voxels in each ROI were averaged. The FC was computed using the Fisher-transformed bivariate correlation coefficients between two ROI BOLD time series. All pairs of ROIs constructed an FC matrix for each subject. We computed the DCs of the VAN, SN, and FPN using graph theory in the same way as in the main analyses.

**Statistical analyses**

We first calculated the differences in DCs between resting-state and oddball task fMRI, focusing on the VAN, SN, and FPN. Next, we investigated the correlations of DCs between the DAN and the other networks during both resting state and the oddball task. Finally, we calculated the correlations between MMI and the DCs during resting state and the oddball task for each of these three networks. Throughout these analyses, a p-value of < 0.05 was considered to indicate a significant difference. Multiple comparison corrections were not applied because of the exploratory nature of these supplementary analyses.

References

1 Loh, K. K. & Kanai, R. Higher media multi-tasking activity is associated with smaller gray-matter density in the anterior cingulate cortex. PLoS One 9, e106698, doi:10.1371/journal.pone.0106698 (2014).

2 Ophir, E., Nass, C. & Wagner, A. D. Cognitive control in media multitaskers. PNAS 106, 15583-15587 (2009).

3 Cole, M. W., Reynolds, J. R., Power, J. D., Repovs, G., Anticevic, A., Braver, T. S. Multi-task connectivity reveals flexible hubs for adaptive task control. Nat Neurosci. 16(9): 1348– 1355. doi:10.1038/nn.3470 (2013).

4 Fox. M.D., Corbetta. M., Snyder. A. Z, Vincent. J. L, Raichle. E. M: Spontaneous neuronal activity distinguishes human dorsal and ventral attention systems. PNAS 103 (26) 10046- 10051; https://doi.org/10.1073/pnas.0604187103 (2006)

5 Yeo, B. T. et al. The organization of the human cerebral cortex estimated by intrinsic functional connectivity. J Neurophysiol 106, 1125-1165, doi:10.1152/jn.00338.2011 (2011).

6 Shirer, W. R., Ryali, S., Rykhlevskaia, E., Menon, V. & Greicius, M. D. Decoding subject-driven cognitive states with whole-brain connectivity patterns. Cereb. Cortex **22**, 158-165, doi:10.1093/cercor/bhr099 (2012).

|  | df | DC mean | | DC SD | | t | Cohen’s  d | p |
| --- | --- | --- | --- | --- | --- | --- | --- | --- |
|  |  | rest | task | rest | task |  |  |  |
| VAN | 103 | 25.90 | 21.04 | 10.06 | 8.27 | 6.22 | 0.61 | < 0.001 |
| SN | 103 | 34.60 | 31.10 | 11.61 | 10.48 | 4.29 | 0.42 | < 0.001 |
| FPN | 103 | 31.62 | 29.12 | 9.18 | 8.51 | 3.38 | 0.33 | 0.001 |

**Table S1. Differences in DCs between resting-state and oddball task fMRI for the VAN, SN, and FPN**

VAN, ventral attention network; SN, salience network; FPN, frontoparietal network; DC, degree centrality; SD, standard deviation; df, degrees of freedom.

|  | df | Correlation  coefficient | p |
| --- | --- | --- | --- |
| Rest VAN | 103 | 0.20 | 0.047 |
| Task VAN | 103 | 0.28 | 0.004 |
| Rest SN | 103 | 0.04 | 0.72 |
| Task SN | 103 | 0.13 | 0.21 |
| Rest FPN | 103 | 0.05 | 0.61 |
| Task FPN | 103 | -0.09 | 0.37 |

**Table S2. Correlations between the DCs of the DAN and other networks during resting state**

VAN, ventral attention network; SN, salience network; FPN, frontoparietal network; df, degrees of freedom.

|  | df | Correlation  coefficient | p |
| --- | --- | --- | --- |
|  |  |  |  |
| Rest VAN | 103 | 0.11 | 0.29 |
| Task VAN | 103 | 0.18 | 0.07 |
| Rest SN | 103 | -0.08 | 0.43 |
| Task SN | 103 | -0.01 | 0.90 |
| Rest FPN | 103 | -0.06 | 0.57 |
| Task FPN | 103 | -0.17 | 0.09 |

**Table S3. Correlations between the Media Multitasking Index and DCs during resting-state fMRI**

VAN, ventral attention network; SN, salience network; FPN, frontoparietal network; df, degrees of freedom.
